# Sexual function after robot-assisted prolapse surgery: a prospective study

**DOI:** 10.1007/s00192-018-3645-z

**Published:** 2018-04-23

**Authors:** Femke van Zanten, Cherèl Brem, Egbert Lenters, Ivo A. M. J. Broeders, Steven E. Schraffordt Koops

**Affiliations:** 1 Department of Gynecology, Meander Medical Center, Maatweg 3, 3813 TZ Amersfoort, The Netherlands; 2 Faculty of Science and Technology, Institute of Technical Medicine, Twente University, Enschede, The Netherlands; 3 Department of Surgery, Meander Medical Center, Amersfoort, The Netherlands

**Keywords:** Pelvic organ prolapse, PISQ-12, Robot-assisted, Sacrocervicopexy, Sacrocolpopexy, Sexual function

## Abstract

**Introduction and hypothesis:**

Female pelvic organ prolapse (POP) can severely influence sexual function. Robot-assisted surgery is increasingly used to treat POP, but studies describing its effect on sexual function are limited. The objective of this study was to evaluate sexual function after robot-assisted POP surgery.

**Methods:**

This prospective cohort study included all patients who underwent a robot-assisted sacrocolpopexy (RASC) or supracervical hysterectomy with sacrocervicopexy (RSHS). Exclusion criteria were unknown preoperative sexual activity status or concomitant surgery. In sexually active women, sexual function was measured with the translated validated version of the Pelvic Organ Prolapse/Urinary Incontinence Sexual Questionnaire (PISQ-12). Changes in sexual activity were scored. Prolapse stages were described using the simplified Pelvic Organ Prolapse Quantification (S-POP) system.

**Results:**

A total of 107 women were included (median follow-up 15.3 months). No difference was found in the total number of sexually active women before and after surgery [63 (58.9%) vs. 61 (63.5%), *p* = 0.999]. Significantly fewer women avoided sexual intercourse postoperatively compared with preoperatively. Preoperatively, sexual intercourse was avoided due to vaginal bulging (2% vs. 24%, respectively, *p* = 0.021). Total mean PISQ-12 scores improved significantly 1 year after prolapse correction (33.5 vs. 37.1; *p* = 0.004), mainly due to improved scores on the physical and behavioral–emotive domain. No significant difference in pre- and postoperative complains of dyspareunia was found.

**Conclusion:**

Robot-assisted middle-compartment surgery improved sexual function 1 year after surgery according to enhanced physical and emotional scores. The total number of sexually active women and complains of dyspareunia before and after surgery did not differ.

## Introduction

An increasing number of women suffer from pelvic organ prolapse (POP) [1]. This condition is known to negatively impact sexual functioning and quality of life (QoL) [2, 3]. Sexual dysfunction as a result from POP can be caused by the sensation of vaginal pressure, dyspareunia, and/or embarrassment during sexual activity. Laparoscopic sacrocolpopexy (LSC) is the gold standard for the treatment of apical/vault prolapse [4]. The open procedure was first reported by Lane in 1962 for women suffering from vaginal vault prolapse following total hysterectomy [5]. In search of a technique in which vaginal function posthysterectomy was not lost, Lane proposed to anchor the prolapsed vaginal vault to the sacral promontory with the use of synthetic material. This led eventually to the abdominal sacrocolpopexy as we know it today. Throughout the years, several studies have shown that sacrocervicopexy following a subtotal hysterectomy is an effective treatment for uterine prolapse [6, 7]. Investigating the influence of these middle-compartment operative prolapse techniques on sexual function, and therefore QoL, is an important issue.

Robot-assisted prolapse surgery is increasingly used due to the technical advantages of the robot. Three-dimensional vision, physiologic tremor filtering, increased freedom of instrument movement, and optimal ergonomics characterize the robot-assisted approach. These factors may help the surgeon perform a deep and precise dissection in the pelvis and anchor the mesh over the prolapsed walls as much as possible in an effort to minimize recurrence and mesh-related complications. Despite the increasing use of robotics for apical prolapse correction, studies describing sexual function after robot-assisted sacrocolpopexy (RASC) are scarce [8–11]. Considering this lack of studies on sexual function after robot-assisted abdominal prolapse surgery, we performed a prospective, large-cohort study using the translated and validated version of the Pelvic Organ Prolapse/Urinary Incontinence Sexual Questionnaire (PISQ-12) as a primary outcome measurement [3].

## Materials and methods

This study is part of the Prospective Assessment of Robotic Sacrocolpopexy: a European Multicentric Cohort (PARSEC) database and a planned ancillary analysis (https://clinicaltrials.gov; identifier NCT01598467). All patients suffering from vaginal middle-compartment prolapse treated with RASC or robot-assisted supracervical hysterectomy with a sacrocervicopexy (RSHS) between 2012 and 2015 were prospectively included. Prolapse stages were described using the simplified Pelvic Organ Prolapse Quantification (S-POP) [12, 13]. The S-POP is a validated short form of the standard POP-Q system. It describes only four vaginal landmarks (anterior and posterior vaginal walls, cervix/vaginal cuff, posterior fornix) instead of nine. Only women with S-POP stage ≥2 (stage 2: given point of the S-POP descends between 1 cm above to 1 cm below the hymenal remnants) were included. Patients with no documented preoperative sexual activity status were excluded. To eliminate the influence of concomitant surgeries on sexual function, patients undergoing concomitant surgery were excluded (anterior and/or posterior colporrphaphy, rectopexy, anti-incontinence surgery, oophorectomy). Patients were considered lost to follow-up if their sexual activity status postoperatively was unknown.

Sexual function was measured by scoring sexual activity status (i.e., sexually active vs. inactive) and by using the translated and validated Dutch version of the Pelvic Organ Prolapse/Urinary Incontinence Sexual Questionnaire (PISQ-12) [3]. The PISQ-12 was given to all patients before and 1 year after surgery. All patients were seen preoperatively in the outpatient clinic and 6 weeks and 1 year after surgery. Patients filled in the PISQ-12 independently, and the questionnaire was handed in during the 1-year clinical evaluation. In case patients refused or were not able to visit the clinic, the questionnaire was sent by post. The PISQ-12 evaluates sexual function in women with urinary incontinence and/or pelvic organ prolapse (POP) [14]. It is the validated short form of the PISQ-31 and therefore more manageable for patients to answer. The PISQ-12 contains 12 questions concerning sexual functioning in three domains: behavioral–emotive (items 1–4), physical (items 5–9), and partner-related (items 10–12) [3]. Five response options were provided (always, mostly, sometimes, seldom, never). The scores of the first four questions ranged from 0 = never to 4 = always, and the last eight questions ranged from 0 = always to 4 = never. The sum of all answers was computed to form a total score (range 0–48), with higher scores indicating better sexual function. Questionnaires with up to two missing answers were accepted, and missing items were replaced with the mean of all answered items. The PISQ-12 is not validated for patients who are sexually inactive. In such cases, this was listed in their medical record to detect changes in sexual activity before and after surgery. To allow for comparison of PISQ-12 scores, only women who filled in the questionnaire both before and after surgery were analyzed.

Each PISQ-12 item was divided to describe percentages, in which answers of “always or mostly” were considered as one group, “sometimes” as a second group, and “seldom or never” as the third group. De novo dyspareunia was defined when dyspareunia preoperatively was scored “seldom or never” and postoperatively “always or mostly.”

Deterioration and improvement ratios according to the PISQ-12 in individual sexually active patients were calculated using the following formula: [(total PISQ-12 score follow-up) – (total PISQ-12 score preoperative)] / (total PISQ-12 score preoperative) × 100. Ratios were considered stable if they changed by <10%, as improved if they increased by 10–50%, very improved if they increased by >50%, worsened if they decreased by 10–50%, and very worsened if they decreased by >50%. Ultimately, separate scores for the two surgical techniques (RASC and RSHS) and for the two different age groups (≤ 60 and > 60) were given, as more recurrent prolapses may be seen in the age group ≤60 years after prolapse surgery [15].

All surgical procedures were performed by experienced gynecologists using the da Vinci® Robot (Intuitive Surgical, Inc., Sunnyvale, CA, USA). The technique used to perform pelvic floor correction was similar to that described by Clifton et al. [16]. In patients with the uterus present, a subtotal hysterectomy was performed. For prolapse correction, macroporous polypropylene mesh (type 1, Prolene, Ethicon Inc., Johnson & Johnson, Hamburg, Germany) was used. The mesh was sutured to the anterior and posterior vaginal wall with nonabsorbable sutures (Ethibond, Ethicon Inc., Johnson & Johnson, Hamburg, Germany) and intracorporeally configured into a *Y* shape. The mesh was anchored proximally to the sacral promontory using titanium tacks (Autosuture Protack 5 mm, Covidien, USA).

This study was carried out in accordance with the ethical standards of The National Central Committee on Research Involving Human Subjects (CCMO). Statistical analysis was performed using IBM SPSS statistics version 22.0. Mean values were compared using the two-sided independent or paired samples *t* test and median values using a Mann–Whitney *U* test or Wilcoxon signed-rank test, as appropriate. Nominal or ordinal data were analyzed with chi-squared, Fisher’s exact, or McNemar tests. An alpha value of 0.05 was considered significant.

## Results

**Fig. 1:**
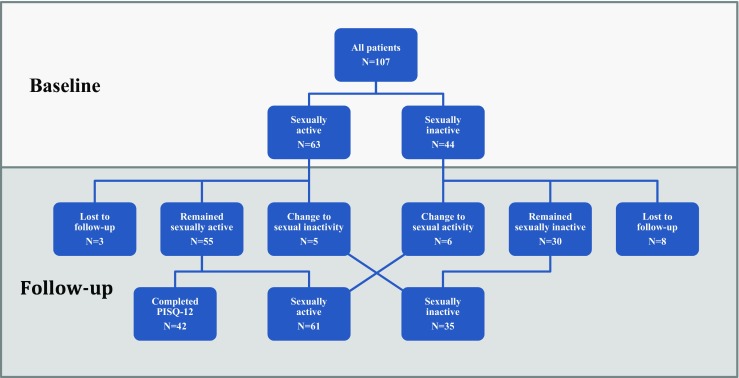
Study participants. *N* number, *PISQ-12* Pelvic Organ Prolapse/Urinary Incontinence Sexual Questionnaire

One hundred and nineteen patients underwent eligible surgery. In 12 patients, sexual activity was unknown at baseline, resulting in a final sample size of 107 patients (Fig. 1). Sixty-three patients (58.9%) were sexually active and 44 patients (41.1%) sexually inactive. Eleven of the 107 patients (10.3%) were lost to follow-up (patient preference because of no complains *N* = 5, incorrect contact details *N* = 1, no reason/other *N* = 5). Of the remaining 96 participants, 92 (95.8%) were seen postoperatively in the outpatient clinic, and four (4.2%) sent in the completed questionnaire by post. The median follow-up duration was 15.3 months (range 8.9–44.4). The baseline characteristics of all patients are listed in Table 1. An RASC was performed on 45 patients (42%; including two conversions to an open abdominal sacrocolpopexy) and an RSHS in 62 (58%) patients. Almost half of all patients (44.9%) had a history of prolapse or anti-incontinence surgery, and 82.2% were postmenopausal. The use of preoperative vaginal estrogens was low (3.7%). Sexually active and inactive patients were compared on various demographic characteristics. These two groups differed in age, postmenopausal status, and mean S-POP C (middle compartment). A more severe prolapse was seen in sexually inactive women (Table 1).

**Table 1:** Baseline patient characteristics

Characteristics	All patients	Sexually active	Sexually inactive	*p* value^a^
	(*n* = 107)	(*n* = 63)	(*n* = 44)	
Age (years), mean ± SD (range)	61.5 **±** 10.6	58.8 **±** 10.1	65.4 **±** 10.2	0.001^b^
BMI (kg/m^2^), median (range)	25.5	25.1	26.8	0.093
	(19.6–37.8)	(19.8–35.5)	(19.6–37.8)	
Parity, median (range)	3.0 (0–7)	2.5 (1–7)	3.0 (0–6)	0.208
Postmenopausal, *N* (%)	88 (82.2)	47 (74.6)	41 (93.2)	0.027^b^
S-POP, mean (range)				
A	2.9 (1–4)	2.8 (1–4)	3.0 (1–4)	0.382
B	2.1 (1–4)	2.0 (1–4)	2.3 (1–4)	0.236
C	2.9 (1–4)	2.7 (1–4)	3.1 (1–4)	0.046^b^
D	2.2 (1–4)	1.9 (1–4)	2.5 (1–4)	0.103
Previous intra-abdominal surgery,^c^ *N* (%)	72 (67.3)	40 (63.5)	32 (72.7)	0.316
Previous prolapse/incontinence surgery, *N* (%)	48 (44.9)	29 (46.0)	19 (43.2)	0.771
Previous hysterectomy, *N* (%)	45 (42.1)	23 (36.5)	22 (50.0)	0.164
Dyspareunia, *N* (%)	N/A	25 (39.7)	N/A	N/A
Symptoms of bulge, *N* (%)	103 (96.3)	61 (96.8)	42 (95.5)	0.999
Preoperative use of vaginal estrogens, *N* (%)	4 (3.7)	1 (1.6)	3 (6.8)	0.303

*SD* standard deviation, *BMI* body mass index, *N/A* not applicable, *S-POP* simplified Pelvic Organ Prolapse Quantification

^a^ Comparing sexually active with sexually inactive

^b^ Statistically significant

^c^ Including vaginal hysterectomy

### Sexual function measured with PISQ-12

Forty-two of the 55 patients (76.4%) who were sexually active both before and after surgery filled in a pre- and postoperative PISQ-12. The other patients were either sexually inactive pre-operatively (*N* = 44) or changed from being sexually active to sexually inactive postoperatively (*N* = 5), and could therefore not complete a PISQ-12. Furthermore, three patients were lost to follow-up (Fig. 1). Total PISQ-12 scores improved significantly from 33.5 to 37.1 (*p* = 0.004). Of the 12 separate items, two showed a significant improvement in orgasm frequency and avoidance of sexual activity because of symptoms of vaginal bulging (Table 2). Both the physical and the behavioral–emotive domain showed overall significant improvement in mean [± standard deviation (SD)] scores (respectively, 2.9 ± 0.9 vs. 3.5 ± 0.6; *p* = 0.003 and 2.5 ± 0.7 vs. 2.8 ± 0.7; *p* = 0.029). No difference was seen in the partner-related domain (2.9 ± 0.6 vs. 2.9 ± 0.6; *p* = 0.804).

**Table 2:** PISQ-12^a^ scores

	Preoperative baseline	1 year after surgery	*p* value
	(*n* = 42)	(*n* = 42)	
1 Sexual desire	2.3 (0.7)	2.4 (0.8)	0.675
2 Orgasm frequency	2.3 (1.1)	2.7 (1.0)	0.044^b^
3: Arousal	2.8 (1.0)	3.0 (0.9)	0.146
4: Satisfaction variety sexual activities	2.7 (1.0)	3.0 (0.9)	0.110
5: Dyspareunia	2.5 (1.5)	2.7 (1.4)	0.391
6: Urinary incontinence during sexual activities	3.3 (1.1)	3.6 (0.9)	0.104
7: Fear of fecal or urinary incontinence during sexual activities	3.3 (1.2)	3.7 (0.7)	0.104
8: Avoidance of sexual activity because of symptoms of vaginal bulging	2.6 (1.4)	3.8 (0.6)	<0.0005^b^
9: Negative emotions during sexual intercourse	3.1 (1.1)	3.6 (0.9)	0.063
10: Erection problem partner	3.4 (0.9)	3.5 (1.0)	0.393
11: Premature ejaculation partner	3.6 (0.7)	3.6 (0.8)	0.762
12: Change in orgasm intensity	1.6 (1.0)	1.7 (0.7)	0.618
Total PISQ-12 score	33.5 (5.6)	37.1 (5.4)	0.004^b^

Data presented as mean (SD)

*PISQ-12* Pelvic Organ Prolapse/Urinary Incontinence Sexual Questionnaire, *SD* standard deviation

^a^Questions of the PISQ-12 are shortened

^b^Statistically significant

The individual questions of the PISQ-12 were converted into percentages, and these results are depicted in Table 3. Preoperatively, almost a quarter of all sexually active women (24%) avoided sex always or mostly because of symptoms of vaginal bulging. This number significantly decreased to 2% 1 year after prolapse correction (*p* = 0.021). Similarly, the number of women suffering from dyspareunia decreased after surgery, from 29 to 17%. Patients who reported to have dyspareunia sometimes increased from 10% to 26%. Item 5, reporting the mean score of dyspareunia, improved but not significantly (Table 2). De novo dyspareunia occurred in two patients (4.7%). One suffered from vaginal atrophy; neither had a mesh exposure. In total, seven patients (6.5%) started using vaginal estrogens 6 weeks after surgery and continued use until follow-up 1 year later. Four patients (3.7%) were advised to start using vaginal estrogens at their 1-year consultation. In total, one patient suffered from a small vaginal mesh exposure. She had no complains of dyspareunia either before or after surgery.

**Table 3:** PISQ-12 items as percentages

	Preoperative baseline (*n* = 42)			1 year after surgery (*n* = 42)			*p*-value^a^
	Always or mostly	Sometimes	Seldom or never	Always or mostly	Sometimes	Seldom or never	
1: Sexual desire	43	45	12	48	38	12	0.999
2: Orgasm frequency (2)	41	38	21	67	21	12	0.063
3: Arousal	62	24	7	79	14	7	0.999
4: Satisfaction variety sexual activities	69	10	14	81	5	12	0.999
5: Dyspareunia	29	10	62	17	26	57	0.687
6: Urinary incontinence during sexual activities	10	12	79	5	5	90	0.687
7: Fear of fecal or urinary incontinence during sexual activities	10	12	79	2	7	88	0.375
8: Avoidance of sexual activity because of symptoms of vaginal bulging	24	21	50	2	2	91	0.021^b^
9: Negative emotions during sexual intercourse	7	21	71	5	7	88	0.999
10: Erection problem partner	2	21	76	10	5	86	0.999
11: Premature ejaculation partner	2	10	88	2	12	83	0.999
12: Change in orgasm intensity	17	31	50	7	60	31	0.999

PISQ-12 questions are shortened

*PISQ-12* Pelvic Organ Prolapse/Urinary Incontinence Sexual Questionnaire

^a^ Comparing preoperative with postoperative “always or mostly” and “seldom or never”

^b^ Statistically significant

After surgery, 48 and 7% of patients’ sexual function was improved and very improved, respectively, and remained stable in 31%. In six women (14%), sexual function worsened; in three of these patients, a recurrent prolapse was diagnosed (Table 4). In subgroup analyses (age and surgery type), no statistically significant differences were seen in deterioration and amelioration of sexual function (Table 4). No difference was seen in total PISQ-12 scores pre- and postoperatively when comparing the two age groups and surgical technique [preoperative: 33.9 (≤60) vs. 32.3 (>60), *p* = 0.343; postoperative: 37.5 (≤60) vs. 37.0 (>60), *p* = 0.741; preoperative: 32.4 (RASC) vs. 33.7 (RSHS), *p* = 0.416; postoperative 37.0 (RASC) vs. 37.5 (RSHS), *p* = 0.726].

**Table 4:** Deterioration and amelioration of total PISQ-12 scores

	All patients	Age ≤ 60	Age > 60	*p*-value^a^	RASC	RSHS	*p*-value^b^
	(*n* = 42)	(*n* = 24)	(*n* = 18)		(*n* = 18)	(*n* = 24)	
Very improved	7.1% (3)	4.2% (1)	11.1% (2)	0.567	16.7% (3)	0% (0)	0.071
Improved	47.6% (20)	54.2% (13)	38.9% (7)	0.327	33.3% (6)	58.3% (14)	0.108
Stable	31.0% (13)	33.3% (8)	27.8% (5)	0.700	33.3% (6)	29.2% (7)	0.773
Worsened	14.3% (6)	8.3% (2)	22.2% (4)	0.375	16.7% (3)	12.5% (3)	0.999
Very worsened	0.0% (0)	0.0% (0)	0.0% (0)	N/A	0.0% (0)	0.0% (0)	N/A

Data presented as % (*N*)

*N/A* not applicable, *PISQ-12* Pelvic Organ Prolapse/Urinary Incontinence Sexual Questionnaire, *RASC* robot-assisted sacrocolpopexy, *RSHS* robot-assisted supracervical hysterectomy with sacrocervicopexy

^a^ Comparing age <60 with >60

^b^ Comparing RASC with RSHS

### Changes in sexual activity status

No significant differences were found in the number of sexually active women before and after surgery [63 (58.9%) vs. 61 (63.5%), *p* = 0.999]. Most women who were sexually active before surgery stayed sexually active after surgery (55/63). Six patients (9.5%) who were sexually inactive before surgery reported being sexually active after prolapse correction. Two of these six patients cited prolapse-related issues as the reason for this change. For the remaining four patients, the source of the change is unknown. Five patients (7.9%) were sexually active before surgery but sexually inactive after surgery. Reasons for this change were loss of their partner (*N* = 2), no more interest in sex (*N* = 1), impossible to have sexual intercourse due to a painful sensation in the vagina since the operation (*N* = 1), and no reason given (*N* = 1).

## Discussion

This prospective study shows a significant improvement in sexual function in patients with POP 1 year after robot-assisted prolapse surgery. Both physical and behavioral–emotive domains of the PISQ-12 improved significantly. This suggests that amelioration of total sexual function was mainly caused by better physical and emotional scores after surgery. Due to the complicated nature of sexual functioning [17], it must be taken into account that more factors could have contributed to the changes observed here. Significantly fewer women avoided sex postoperatively compared with preoperatively due to vaginal bulging. However, the total number of patients suffering from dyspareunia did not significantly change. Postoperatively, fewer patients reported having dyspareunia always or mostly. De novo dyspareunia occurred in 4.7% of patients. No differences were seen in the total number of sexually active women before and after surgery, and there was no discernible trend in the reasons for changes in sexual activity. Additionally, the amount of improvement of sexual function in women >60 years appeared to be similar to that in younger women.

A more severe prolapse of the middle compartment (S-POP C) was found in sexually inactive women. This implies that severity of apical prolapse and sexual function are directly related, which is in line with Ellerkmann et al. [18]. Another explanation could be that participants who are sexually active seek medical help at a less severe stage of prolapse. There was no difference in the use of vaginal estrogens between sexually active and inactive patients. Vaginal estrogens were only prescribed in case of complains (such as vaginal dryness or dyspareunia) and not as a preventive measure. Some research indicates that intravaginal estrogens may improve symptoms of vaginal atrophy in postmenopausal patients, but the evidence is not conclusive [19]. Only a very small percentage of all patients in this study used vaginal estrogens, and therefore, we believe the influence on the results will be limited.

The robot-assisted technique may be of influence in the improvement of sexual function. Women with POP may have decreased vaginal wall sensitivity [20], and surgical correction could possibly play a role in the restoration of clitoral and vaginal wall sensation [21]. The robot-assisted approach is associated with improved performance of intracorporeal suturing compared with laparoscopic surgery [22]. Precise suturing of the vaginal walls could benefit sexual function. A randomized controlled trial with adequate power is the only way to resolve this question. Only a few other studies with limited patients have described sexual function after using robot-assisted prolapse surgery [8–11]. One study performed by Geller et al. [8] investigated outcomes 1 year after RASC in 25 women with advanced POP and showed stable sexual function but no improvement after surgery. One randomized trial [23] compared LSC with RASC and showed an improvement in total PISQ-12 scores for both groups. The number of sexually active patients postoperatively was 14 and 15 in the two groups. Prior published articles after LSC have documented an improvement in sexual function [24–28], which is in line with our results.

Strong points of this study are the prospective design, the large number of patients included, and the long median follow-up (15.3 months). Heterogeneity of the study group was minimized by standardized surgery and by only evaluating patients after a RASC or RSHS. Patients who underwent concomitant vaginal surgery were excluded, as this may lead to vaginal narrowing and possibly dyspareunia [29]. Anti-incontinence procedures were also eliminated. The latter is thought to compromise neural tissue in the vaginal wall, also known as the female prostate, thereby affecting orgasmic response [30].

Limitations of this study are that patients were treated in a tertiary referral hospital for pelvic floor dysfunction. Therefore, this population often had a history of pelvic floor disorders, which could limit the generalizability of the study. Procedures were performed by surgeons experienced in open abdominal, conventional laparoscopic, and robot-assisted pelvic prolapse procedures. This is important, because tensioning of the mesh is a crucial aspect of this procedure. Using too little or too much tension may result in either insufficient repair or postoperative pain or discomfort [16]. Second, only one center was included in this study. Third, we did not conduct a randomized controlled trial comparing robot-assisted surgery with other techniques. Finally, not all sexually active patients filled in the questionnaire, resulting in loss to follow-up. Although all patients received the questionnaire, some left it blank or only partly completed it. Possible reasons are negative feelings, such as shame regarding questions about their sexual life, or due to different cultural aspects.

Patients who underwent a subtotal hysterectomy concomitantly were not excluded, since this is an essential part of treating apical prolapse. A hysterectomy may affect sexual function negatively (absence of rhythmic uterine contractions during orgasms) or positively (resolving underlying uterine condition such as prolapse, menorrhagia) [29]. Zucchi et al. [31] found an improvement of sexual function after removal of the uterus with prolapse surgery. We found no difference in pre- and postoperative sexual function between the two surgery types. However, our finding was based on subgroup analysis with a limited number of patients (*N* = 24 and *N* = 18), making further research necessary.

In spite of the worldwide increased use of robot-assisted prolapse surgery [32], data on sexual function after the robotic technique are limited. The results of this study contribute to a better knowledge of sexual dysfunction in patients suffering from POP treated with the robot-assisted approach.

## Conclusion

This prospective cohort study shows improvement in sexual function 1 year after robot-assisted apical prolapse surgery due to enhanced physical and emotional scores. The total number of sexually active women and women suffering from dyspareunia before and after surgery did not differ. The findings are important for proper counselling of patients prior to operative POP treatment.
